# Corporate social responsibility, debt financing cost and enterprise innovation

**DOI:** 10.1038/s41598-022-26076-3

**Published:** 2022-12-19

**Authors:** Jing-jing Yao, Yi-ang Qi, Bing Guo

**Affiliations:** School of Finance and Economics, Jiangsu University, Zhenjiang, China

**Keywords:** Scientific data, Statistics, Socioeconomic scenarios

## Abstract

Based on the data of A-share listed companies in China from 2016 to 2020, this study empirically analyzes the relationship among corporate social responsibility, debt financing cost and enterprise innovation by constructing a regulated mediation effect model. The results show that enterprises perform social responsibility actively can enhance the level of enterprise innovation. Besides, debt financing cost plays a part of the intermediary role between corporate social responsibility and enterprise innovation. It is found that market competition degree positively regulates the relationship between corporate social responsibility and enterprise innovation, and market competition degree strengthens the part of the intermediary effect of debt financing cost. The conclusions not only help to reveal the impact mechanism of corporate social responsibility on enterprise innovation, but also provide empirical evidence for promoting enterprises to actively assume social responsibility, improve the level of innovation, and provide empirical evidence for the government to formulate corresponding policies according to the degree of competition in different markets.

## Introduction

As the basic unit of the national economy, the innovation ability of enterprises has an important impact on the enterprise itself, the industry and region, and even at the national level. Nowadays, the innovation level of Chinese enterprises has been greatly improved, and high-tech enterprises such as Huawei, Tencent, and Alibaba have emerged, but these enterprises still have a large room for growth compared with high-tech enterprises in European and American countries, such as Apple Inc. and Microsoft. Besides, with the rapid development of the world economy, food safety, environmental protection and other issues have increasingly entered into public eyes, and corporate social responsibility has begun to be valued. Enterprises themselves are also aware of the necessity of social responsibility, and more and more companies have begun to assume social responsibility and disclose social responsibility reports. Fulfilling social responsibility requires companies to innovate. For example, when meeting the needs of stakeholders for green products, companies will carry out green innovation to reduce the environmental pollution caused by products in the production process.

R&D requires enterprises to invest more human, material and financial resources. Internal funds are usually used for daily business turnover, and most companies are not enough to support innovation if they only rely on internal funds, so the innovation activities of enterprises mainly rely on external funds. Among the external funds, debt financing is the most important source, so the level of debt financing costs has a greater impact on enterprise innovation. However, due to the long enterprise innovation cycle, external investors are usually unwilling to provide debt financing for enterprise innovation at a lower cost. Besides, enterprises, especially small and medium-sized enterprises are faced with financing constraints, which further increases the financing cost. Companies with a limited number of listing years and smaller scales are more constrained by finance and have higher external financing costs, which adversely affects the innovation activities of enterprises. In regions and countries with more developed stock markets, the level of innovation of high-tech industries and industries that rely more on external financing is much higher than that of regions and countries with less developed stock markets. Hence, the development of credit markets has become an important factor affecting the level of innovation in these industries^1^.

Enterprises in many developing countries, like Chinese enterprises are in the stage of economic transformation, and the trend of free competition in various industries is becoming more and more obvious. In the face of different market competition, enterprises often adopt different investment strategies. Fierce market competition has higher requirements for the strength of enterprises, and need enterprises to carry out more research and development innovation.

In summary, this paper selects China's A-share listed companies over the period 2016 to 2020 as a research sample, empirically analyzes the relationship between corporate social responsibility, debt financing cost and enterprise innovation, and examines the mediating effect of debt financing cost and the moderating effect of market competition. Since the relevant research pays less attention to the financing cost, this paper focuses on the debt financing cost according to the main financing method in Chinese enterprises, and discusses the relationship between CSR, debt financing cost and enterprise innovation, which enriches both domestic and foreign research on related aspects. Different from existing research, the contribution of this paper is presented as follows: First, at the theoretical level, the research of Chinese scholars on corporate financing mainly focuses on financing constraints, financing structure, financing methods, etc., however, the literature on financing costs is slightly insufficient. Taking financing cost as the starting point, this paper explores the intermediary effect of debt financing cost between corporate social responsibility and enterprise innovation, and studies the regulatory effect of market competition on the intermediary role so as to enrich the research on financing cost.

Second, at the application level, for enterprises, it is helpful for enterprises to assume social responsibility according to their own market environment, and actively disclose the positive impact of social responsibility information on the company so as to promote corporate social responsibility performance, further help enterprises reduce debt financing cost, and ultimately provide financial support for enterprise innovation; for the government, the adjustment effect of market competition can support the government to formulate corresponding policy decisions according to different market competition. Thus promoting the healthy development of enterprises under different market competition levels.

The article proceeds as follows. Section “Literature review and research hypotheses” covers the theoretical analysis and research hypothesis. Section “Methodology” is our research design, in which we process the sample data, describe the variables, and set the empirical models. Section “Empirical results and discussion” presents the empirical analysis, including descriptive statistics, correlation analysis, regression analysis, and robustness tests. Section “Conclusions and implications” provides the conclusions and policy implications of our research.

## Literature review and research hypotheses

### Corporate social responsibility and enterprise innovation

Stakeholder theory defines the scope of corporate social responsibility. Enterprises can not only value the interests of shareholders, but also assume social responsibility to consumers, employees, suppliers, etc^2,3^. The resources paid by corporate social responsibility can be returned to enterprises in different forms, specifically: First, more and more consumers recognize the importance of environmental protection and their physical health, and begin to take the initiative to pay attention to the situation of corporate social responsibility, so corporate social responsibility can establish a good image of enterprises in the minds of consumers, enhance brand efficiency, and increase corporate reputation^4^. Managers who wish to contribute more to society in the long term should pay attention to their environmental, social, and financial CSR^5,6^. In order to maintain a good reputation among consumers and meet the consumer needs of consumers, enterprises will pay more attention to the quality of their own products and increase innovation^7,8^. Second, corporate social responsibility can bring a high-quality working environment and a fair promotion method for R&D employees, and R&D employees are more willing to show their talents in this treatment in exchange for the recognition of the organization^9^. This exchange can improve the sense of organizational identity and pride of the R&D employees of the enterprise, and further motivate them to develop their own innovation ability and tap their own innovation potential^10^. Finally, fulfilling social responsibility can deepen the information exchange between enterprises and stakeholders, build a more comprehensive and in-depth interactive network, and help enterprises have easier access to external information^11–13^. Through the integration of external information and internal information, enterprises can obtain richer knowledge and information related to innovation, thus providing strong support for enterprises to improve their innovation capabilities^14,15^. In view of the above arguments, we propose:

#### Hypothesis H1

Corporate social responsibility has a positive impact on enterprise innovation.

### The mediating effect of debt financing cost

Based on above assumptions, corporate social responsibility performance can bring many benefits to enterprises, such as attracting new employees, giving enterprises access to long-term debts^16^ and promoting management to focus on the long-term development of enterprises, thereby promoting the improvement of enterprise innovation level^17^. Among many mechanisms in which social responsibility affects enterprise innovation, debt financing cost is likely to be one of the paths^18–22^. Fulfilling social responsibilities brings a good reputation to enterprises, reduces information asymmetry, and obtains political connections to reduce debt financing cost for enterprises, thereby alleviating the higher financial pressure brought by R&D innovation for enterprises^23,24^. More specifically, socially responsible companies have a longer debt maturity than other companies^25–27^. In other words, CSR facilities firms' access to long-term debts. Besides, companies with better corporate social performance are more attractive to lenders when it comes to granting leverage^28,29^. Under the condition of low debt financing cost, sufficient cash flow inflow into the enterprise becomes possible, considering the long-term development of the enterprise in the future, while ensuring the daily operation and production activities of the enterprise, the enterprise will invest in R&D innovation^30^. On the other hand, corporate social responsibility fulfillment and innovative research are investment activities with long-term benefits, and corporate social responsibility can improve the attention and understanding of external investors to such activities, so it is easier for enterprises that fulfill social responsibility to support external investors for their own innovative activities, so as to find a lower source of debt financing for innovation. Therefore, based on the above analysis,we propose the following assumptions:

#### Hypothesis H2

The association between corporate social responsibility and enterprise innovation is mediated by debt financing cost.

### The moderating effect of market competition

In order to maintain the reputation brought by social responsibility, enterprises will carry out innovative research and development, while corporate social responsibility can attract high-quality R&D employees, build a wide network of stakeholder interaction, and promote enterprise innovation from the aspects of corporate strategy and management. When the market competition is high, enterprises need to maintain the reputation brought by fulfilling social responsibilities among consumers, so as to have an advantage in competition with other enterprises, therefore, these enterprises will further increase innovation efforts to meet the needs of the public. Moreover, the core competitiveness requirements of enterprises increase with the increase of market competition, at this time enterprises are more willing to attract excellent innovative talents by fulfilling their social responsibilities, increase the salary and welfare of R&D employees, improve the sense of corporate identity and belonging of R&D employees, stimulate the enthusiasm of such employees, and ensure the innovation vitality and innovation level of enterprises, so as to survive in the fierce market competition^31,32^. Market competition can also promote the circulation of information between markets, in the environment of high market competition, enterprises use the stakeholder interaction network to obtain information related to innovation more efficiently, thereby increasing the positive effect of social responsibility for enterprises.

Corporate social responsibility can reduce debt financing cost and thus promote enterprise innovation, so this paper judges that market competition degree also has a regulatory effect on the intermediary effect of debt financing cost^33,34^. When the market competition is high, enterprises can get more returns to fulfill their social responsibilities, and reduce debt financing cost more effectively. In order to further improve the core competitiveness of the enterprise itself, enterprise is more willing to reduce debt financing cost for enterprise to bring benefits into enterprise innovation, such as directly investing the funds obtained from financing into innovation or other high-value projects, and investing project proceeds into innovation^35,36^. When the market competition is low, it usually indicates that there are monopoly enterprises in the industry, such enterprises usually have more abundant resources to innovate, even if debt financing cost is reduced through social responsibility, and the demand for converting low-cost debt financing into enterprise innovation is relatively low^37^. Therefore, our hypotheses are expressed as below:

#### Hypothesis H3a

Market competition will positively moderate the relationship between corporate social responsibility and enterprise innovation.

#### Hypothesis H3b

Market competition degree will strengthen the intermediary role of debt financing cost in the relationship between corporate social responsibility and enterprise innovation, that is, there is a regulated intermediary effect.

**Figure 1:**
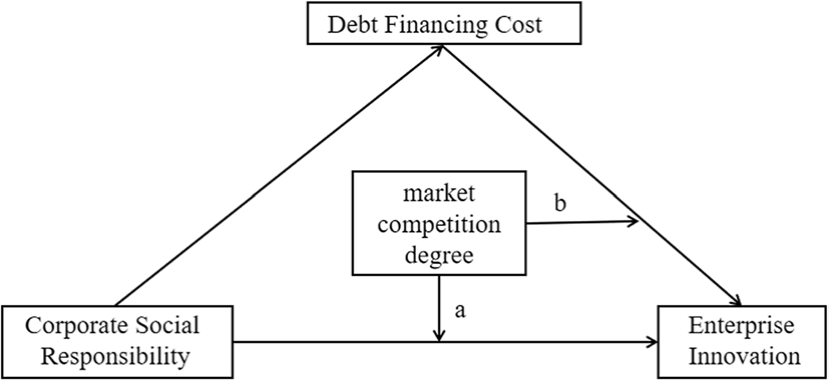
Conceptual framework of the research.

Based on the above analysis, we propose the conceptual body of this paper, as shown in Fig. 1.

Among them, Corporate Social Responsibility is the core independent variable, Enterprise Innovation is the dependent variable, Debt Financing Cost is the mediating variable, and Market Competition Degree is the moderating variable. In Fig. 1, the role of market competitiveness on the mediating effect of the direct path, and the second half path are represented by a and b, respectively.

## Methodology

### Samples and data collection

Our sample consists of China’s A-share listed companies in Shanghai and Shenzhen exchanges from 2016 to 2020. We screen our sample data in four steps. First, we exclude financial and insurance firms with special capital structures. Second, we remove ST and ST* corporations due to the low authenticity and stability. Third, we omit companies with missing key variables. Fourth, to minimize the influence of outliers, we perform tail-reduction processing for all continuous variables below and above the 1% and 99% percentiles, respectively. We finally obtain 11,157 samples. Among them, the corporate interest expense is gathered from the Wind database, the social responsibility comes from the Hexun network, and other data are from the China Stock Market and the Accounting Research (CSMAR) database.

### Variable measures

#### Dependent variable

Enterprise innovation is usually divided into two stages: innovation input and innovation output, but the innovation output is often longer and the uncertainty is higher. So it is difficult to accurately reflect the innovation level of the enterprise in the current period with innovation output. Therefore this paper focuses on considering the enterprise innovation input. Referring to existing research, we measure enterprise innovation (RD) by the ratio of R&D investment in the current period to total assets at the end of the period.

#### Independent variable

Hexun network decomposes corporate social responsibility into five blocks: shareholder responsibility, employee responsibility, supplier, customer and consumer rights responsibility, environmental responsibility, and community responsibility, and sets up multiple indicators under these five blocks. The performance of corporate social responsibility is then scored according to each refined indicator, and finally summed according to a certain weight. The total score is 100, and the higher the score, the better the company's social responsibility. Therefore, this article measures corporate social responsibility by Hexun's score on corporate social responsibility. In addition, to control the effect of the difference on regression, this paper measures corporate social responsibility (CSR) by dividing the score by 100.

#### Mediating variable

The main financing method of Chinese enterprises is debt financing, so the research on financing cost in this paper focuses on debt financing cost, and the ratio of corporate interest expenses to total debt at the end of the period is used to measure the cost of corporate debt financing (COST).

#### Moderating variable

Based on the assets of the enterprise, this paper measures market competition degree by the industry concentration of the enterprise, and the specific calculation method is:

$$ HHI_{j} = - \sum\nolimits_{i = 1}^{n} {\left( {\frac{{x_{ij} }}{{x_{j} }}} \right)}^{2} $$

Among them, x_ij_ denotes the total asset value of i enterprises in the j industry, and x_j_ denotes the sum of the assets of all enterprises in the industry j. First, the ratio of the assets of each enterprise in the industry to the total assets of the industry is calculated, then its square is taken and summed to obtain the industry concentration index. The larger the indicator, the more concentrated the industry, indicating that there are one or more large-scale monopoly enterprises in the industry, and the market competition is small. For the convenience of analysis, this paper takes the negative number of the industry concentration index to measure the market competition degree (HHI).

#### Control variables

In this paper, we select the financial slack (FS), leverage (LEV), age to market (AGE), equity concentration (FSHR), enterprise value (TQ), growth (GROW), return on assets (ROA), enterprise size (SIZE), and nature of ownership (SOE) as the control variables in the empirical research from the perspective of firm characteristics. Besides, based on the results of the Hausman test, a fixed-effect model is used in this paper. Therefore, industry-year fixed effects capture the available information related to each industry in a given year. The variables are defined as shown in Table 1.

**Table 1:** Definition and description of control variables.

Variable type	Variable symbol	Definition and description
Dependent variable	RD	The ratio of the R&D investment of the enterprise to the total assets at the end of the period
Independent variable	CSR	Hexun Network scored Corporate Social Responsibility/100
Mediating variable	COST	The ratio of interest expense for the current period to total liabilities at the end of the period
Adjustment variable	HHI	The opposite of the concentration of the industry in which the enterprise is located
Control variables	FS	The ratio of cash and cash equivalents to total assets
	LEV	The ratio of a company’s total liabilities to total assets at the end of the period
	AGE	Observation year—the year in which the enterprise is listed
	FSHR	The ratio of number of shares held by the largest shareholder to total number of shares
	TQ	The ratio of enterprise Market Value to Total Assets
	GROW	(Current period operating income—previous period operating income)/previous period operating income
	ROA	The ratio of net profit to total assets at the end of the period
	SIZE	Natural logarithm of total assets at the end of the period
	SOE	State-owned companies are equal to 1, and private companies are equal to 0
	YEAR	Annual dummy variable
	IND	Industry dummy variable

### Model setting

Compared to structural equation model, regression analysis is more suitable for studying the specific mechanism of action in the model. And research on regression analysis as an empirical method has been published in many mainstream academic journals in the world. Therefore, we choose regression analysis as an empirical method in this paper. Based on the theoretical analysis, we estimate the following models to verify the research hypotheses proposed above:

First, in order to verify H1, we construct model (1), which takes RD as the dependent variable and CSR as the independent variable.

1 $$ RD = \alpha_{0} + \alpha_{1} CSR + \sum {Controls} + \varepsilon $$

Second, in order to verify H2, we refer to the stepwise method proposed by Baron and Kenny (1986) to test the intermediary role of COST, and build model (2) with COST as the dependent variable and CSR as the independent variable. Besides, on the basis of model (1) and model (2), we also build model (3) in which RD is the dependent variable and CSR is the independent variable.

2 $$ COST = \beta_{0} + \beta_{1} CSR + \sum {Controls} + \varepsilon $$

3 $$ RD = \gamma_{0} + \gamma_{1} CSR + \gamma_{2} COST + \sum {Controls} + \varepsilon $$

First of all, the coefficient $\alpha_{1}$ in model (1), the coefficient $\beta_{1}$ in model (2) and the coefficient $\gamma_{2}$ in model (3) are verified sequentially, if all three coefficients are significant, the intermediary effect of COST is significant, otherwise the bootstrap method is used for revalidation. Then we verify the coefficient $\gamma_{1}$ in model (3), if it is significant, it means that the direct effect between CSR and RD is also significant, otherwise only the intermediary effect is established. The last comparison is the signs between $\gamma_{1}$ and $\beta_{1} \gamma_{2}$, if the two are the same number, it is considered that COST plays a part of the role of intermediary and the proportion of the intermediary effect is ${{\beta_{1} \gamma_{2} } \mathord{\left/ {\vphantom {{\beta_{1} \gamma_{2} } {\alpha_{1} }}} \right. \kern-\nulldelimiterspace} {\alpha_{1} }}$; if the two are different, it is considered to be the masking effect, and the proportion of the intermediary effect is $\left| {{{\beta_{1} \gamma_{2} } \mathord{\left/ {\vphantom {{\beta_{1} \gamma_{2} } {\gamma_{1} }}} \right. \kern-\nulldelimiterspace} {\gamma_{1} }}} \right|$ at this time.

Third, in order to verify H3a, on the basis of model (1), we add HHI and the interaction term CSR*HHI to construct model (4) to test whether the direct effect between CSR and RD is affected by adjustment variable when COST is not considered; If the coefficient $a_{3}$ of interaction term is significant, it means that the adjustment effect of HHI is significant.

4 $$  RD = a_{0}  + a_{1} CSR + a_{2} HHI + a_{3} CSR*HHI + \sum {Controls}  + \varepsilon   $$

In order to verify H3b, we construct a moderated mediating effect model, as shown in model (5) and model (6). If the coefficient $b_{3}$ of CSR*HHI in model (5) and the coefficient $c_{4}$ of COST in model (6) are not 0 at the same time, then HHI moderates the first half path of the intermediary effect; if the coefficient $b_{1}$ of CSR in model (5) and the coefficient $c_{5}$ of COST*HHI in model (6) are not 0 at the same time, then HHI moderates the second half path of the intermediary effect; if the coefficient $b_{3}$ of CSR*HHI in model (5) and the coefficient $c_{5}$ of COST*HHI in model (6) are not 0 at the same time, then HHI moderates both the first half path and the second half path of the mediation effect.

5 $$  COST = b_{0}  + b_{1} CSR + b_{2} HHI + b_{3} CSR*HHI + \sum {Controls}  + \varepsilon   $$

6 $$ RD = c_{0}  + c_{1} CSR + c_{2} HHI + c_{3} CSR*HHI + c_{4} COST + c_{5} COST*HHI + \sum {Controls}  + \varepsilon   $$

## Empirical results and discussion

### Descriptive statistics

Table 2 provides descriptive statistics. From Table 2, it can be seen that the minimum value of RD is 0.0001 and the maximum value is 0.1082, indicating that there is a large gap between the innovation level of different enterprises; the mean and median are 0.0235 and 0.0201 respectively, indicating that the innovation level of Chinese enterprises still has room to rise. The maximum value of CSR is 0.6612, and the mean and median values are 0.2069 and 0.2104 respectively, indicating that the level of corporate social responsibility in China is not high in general, and it is necessary to enhance the willingness and ability to assume social responsibility. The maximum value of COST is 0.055 and the minimum value is 0, indicating that there is a large difference in debt financing cost between enterprises. The maximum value of HHI is − 0.0119, and the minimum value is − 0.2086, indicating that there is a large disparity in the market competition faced by enterprises, and the mean and median are − 0.0554 and − 0.0409, which is very close to 0, indicating that most enterprises are facing fierce market competition.

**Table 2:** Descriptive statistics of variables.

Variables	N	Mean	SD	Min	Median	Max
RD	10,953	0.0235	0.0196	0.0001	0.0201	0.1082
CSR	10,953	0.2069	0.1037	− 0.0337	0.2104	0.6612
COST	10,953	0.0168	0.0129	0	0.0153	0.0550
HHI	10,953	− 0.0554	0.0437	− 0.2086	− 0.0409	− 0.0119
FS	10,953	0.1643	0.1050	0.0148	0.1391	0.6179
LEV	10,953	0.4173	0.1859	0.0741	0.4108	0.8645
AGE	10,953	10.6153	7.3507	0.9699	8.7397	26.9973
FSHR	10,953	0.4796	0.1486	0.1766	0.4733	0.8503
TQ	10,953	1.9222	1.0976	0.8435	1.5851	7.2205
GROW	10,953	0.1802	0.3733	− 0.4761	0.1151	2.3301
ROA	10,953	0.0380	0.0624	− 0.2981	0.0381	0.1905
SIZE	10,953	22.3415	1.2713	20.1193	22.1655	26.3653
SOE	10,953	0.2660	0.4419	0	0	1

### Correlation analysis

Table 3 provides correlation analysis. The correlation coefficient between CSR and RD was 0.023, which is significantly positive at the 5% level, so H1 is preliminarily verified. At the same time, the control variables are significantly related to RD, indicating that the selection of control variables is meaningful. In addition, in order to prevent possible multicollinearity, the variance inflation factor (VIF) of each variable is calculated. The results show that the maximum VIF of all independent variables is only 2.13, which can exclude the influence of multicollinearity. That means there is no high correlation between the explanatory variables in this paper.

**Table 3:** Correlation analysis of variables.

Variables	RD	CSR	COST	HHI	FS	LEV	AGE
RD	1						
CSR	0.023**	1					
COST	− 0.243***	− 0.203***	1				
HHI	0.255***	0.00200	− 0.085***	1			
FS	0.168***	0.154***	− 0.326***	0.069***	1		
LEV	− 0.205***	− 0.125***	0.365***	− 0.131***	− 0.246***	1	
AGE	− 0.235***	− 0.00600	0.189***	− 0.083***	− 0.071***	0.302***	1
FSHR	− 0.101***	0.176***	− 0.153***	− 0.107***	0.057***	0.020**	− 0.148***
TQ	0.277***	0.056***	− 0.170***	0.134***	0.173***	− 0.303***	− 0.131***
GROW	0.022**	0.139***	− 0.073***	0.020**	− 0.00400	0.040***	− 0.085***
ROA	0.123***	0.574***	− 0.275***	0.038***	0.181***	− 0.298***	− 0.119***
SIZE	− 0.243***	0.187***	0.204***	− 0.155***	− 0.133***	0.519***	0.454***
SOE	− 0.187***	0.040***	0.057***	− 0.116***	− 0.024**	0.257***	0.479***

Variables	FSHR	TQ	GROW	ROA	SIZE	SOE	
FSHR	1						
TQ	− 0.072***	1					
GROW	0.039***	0.047***	1				
ROA	0.177***	0.189***	0.228***	1			
SIZE	0.163***	− 0.374***	0.035***	0.0130	1		
SOE	0.136***	− 0.142***	− 0.048***	− 0.064***	0.360***	1	

***, ** and * respectively indicate that the parameter estimation is significant at the levels of 0.01, 0.05 and 0.1.

### Regression analysis

#### Basic result

The regression result of CSR to RD in this paper are shown in Column (1) of Table 4, and the regression coefficient of CSR is 0.0119, which is significantly positive at the level of 1%, indicating that corporate social responsibility has promoted the improvement of enterprise innovation. Therefore, the result of the main regression analysis shows that good corporate social responsibility performance helps enterprises to increase investment in innovation, verifying our research H1.

**Table 4:** Corporate social responsibility, debt financing cost and enterprise innovation.

Variables	Model 1	Model 2	Model 3
	RD	COST	RD
CSR	0.0119*** (6.03)	− 0.0115*** (− 8.62)	0.0098*** (5.00)
COST			− 0.1787*** (− 12.79)
FS	0.0115*** (7.38)	− 0.0225*** (− 21.23)	0.0075*** (4.74)
LEV	0.0010 (0.87)	0.0191*** (25.68)	0.0044*** (3.91)
AGE	− 0.0003*** (− 11.39)	0.0001*** (3.40)	− 0.0003*** (− 11.05)
FSHR	− 0.0056*** (− 4.88)	− 0.0116*** (− 14.97)	− 0.0076*** (− 6.68)
TQ	0.0035*** (21.62)	− 0.0002* (− 1.89)	0.0035*** (21.55)
GROW	− 0.0005 (− 1.26)	− 0.0016*** (− 5.62)	− 0.0008* (− 1.95)
ROA	0.0160*** (4.81)	− 0.0184*** (− 8.16)	0.0127*** (3.84)
SIZE	− 0.0002 (− 0.92)	0.0008*** (6.66)	− 0.0000 (− 0.11)
SOE	− 0.0000 (− 0.07)	− 0.0025*** (− 8.74)	− 0.0005 (− 1.14)
Constant	0.0012 (0.31)	0.0104*** (3.89)	0.0031 (0.79)
Observations	10,953	10,953	10,953
R-squared	0.351	0.300	0.361
Year FE	YES	YES	YES
Industry FE	YES	YES	YES

***, ** and * respectively indicate that the parameter estimation is significant at the levels of 0.01, 0.05 and 0.1. The “t” value is in parentheses.

#### Mediating effect analysis

The test of the mediating effect uses the stepwise regression method, and H2 is tested by model 1, model 2 and model 3. From columns (1)–(3) of Table 4, it can be seen that the coefficient of CSR in the model (1), the coefficient of CSR in model 2 and the coefficient of COST in the model 3 are all significant at the level of 1%, indicating that COST plays an intermediary role in the relationship between CSR and RD. As shown in Table 4 (3), the coefficient of CSR in the model 3 is significantly positive at the level of 1%, that means the direct effect is significant and COST plays a part of the intermediary role between CSR and RD. Finally, combined with the regression coefficients of each model, the indirect effect of COST is 0.0021 ($\beta_{1} \gamma_{2}$), which is the same as the sign of direct effect 0.0098. It fully proves that COST is part of the intermediary factor of CSR influencing RD, and the proportion of intermediary effect to total effect is 17.27% (${{\beta_{1} \gamma_{2} } \mathord{\left/ {\vphantom {{\beta_{1} \gamma_{2} } {\alpha_{1} }}} \right. \kern-\nulldelimiterspace} {\alpha_{1} }}$). Fulfilling social responsibilities brings a good reputation to enterprises, reduces information asymmetry, and obtains political connections to reduce debt financing costs for enterprises, thereby alleviating the higher financial pressure brought by R&D innovation for enterprises. Consequently, the above results support H2: that corporate social responsibility positively relate to enterprise innovation, with debt financing cost partially mediating this relationship.

#### Moderating effect analysis

The moderating effect of market competition on the main effect is shown in Column (1) of Table 5, and the coefficient of CSR*HHI in the model 4 is significantly positive at the level of 1%, indicating that HHI positively moderates the correlation between CSR and RD, and the greater HHI, the stronger the positive effect of CSR on RD. The results above verify H3a.

**Table 5:** The moderation effect of market competition degree.

Variables	Model 4	Model 5	Model 6
	RD	COST	RD
CSR	0.0182***	− 0.0136***	0.0145***
	(6.28)	(− 6.86)	(4.99)
HHI	0.0463***	− 0.0048	0.0692***
	(5.59)	(− 0.85)	(6.77)
CSRHHI	0.1092***	− 0.0348	0.0831**
	(3.20)	(− 1.48)	(2.42)
COST			− 0.2322***
			(− 11.08)
COSTHHI			− 1.0816***
			(− 3.91)
FS	0.0109***	− 0.0224***	0.0071***
	(7.08)	(− 21.13)	(4.52)
LEV	0.0013	0.0190***	0.0046***
	(1.22)	(25.61)	(4.15)
AGE	− 0.0003***	0.0001***	− 0.0003***
	(− 10.92)	(3.26)	(− 10.55)
FSHR	− 0.0043***	− 0.0118***	− 0.0062***
	(− 3.85)	(− 15.22)	(− 5.50)
TQ	0.0033***	− 0.0002	0.0033***
	(20.40)	(− 1.53)	(20.38)
GROW	− 0.0008*	− 0.0016***	− 0.0011**
	(− 1.80)	(− 5.48)	(− 2.51)
ROA	0.0158***	− 0.0182***	0.0126***
	(4.78)	(− 8.03)	(3.82)
SIZE	− 0.0001	0.0008***	0.0000
	(− 0.60)	(6.57)	(0.21)
SOE	− 0.0000	− 0.0025***	− 0.0005
	(− 0.09)	(− 8.75)	(− 1.19)
Constant	0.0060	0.0098***	0.0087**
	(1.52)	(3.62)	(2.23)
Observations	10,953	10,953	10,953
R-squared	0.365	0.301	0.374
Year FE	YES	YES	YES
Industry FE	YES	YES	YES

***, ** and * respectively indicate that the parameter estimation is significant at the levels of 0.01, 0.05 and 0.1. The “t” value is in parentheses.

Columns (2) and (3) of Table 5 report the moderating effect of HHI on the mediating effect of COST. The coefficients of CSR, CSR*HHI in the model 5 and COST, COST*HHI in the model 6 are examined sequentially. The above four coefficients are significant at the level of 1%, so the market competition degree adjusts both the first half path and the second half path of the intermediary effect at the same time. When the degree of market competition is high, enterprises can get more returns to fulfill their social responsibilities, reduce the cost of debt financing more effectively, in order to further improve the core competitiveness of enterprises, enterprises are more willing to reduce the cost of debt financing for enterprises to bring benefits into enterprise innovation, such as the financing of funds directly into innovation or into other high-value projects, and the project proceeds into innovation. When the market competition is low, it usually indicates that there are monopoly forces in the industry, such enterprises usually have rich resources to innovate, even if the cost of debt financing is reduced through the fulfillment of social responsibilities, and the demand for converting low-cost debt financing into enterprise innovation is relatively low. It can be concluded that with the increase of market competition, part of the intermediary effect of debt financing cost is gradually enhanced. Therefore, H3b is verified.

### Robustness tests

#### Substitution variable method

This paper selects the ratio of R&D expenditure to enterprise operating income to measure the innovation investment level of enterprises, and the regression results are shown in Table 6. It can be seen that CSR and RD in model 1 are significantly positively correlated at the 1% level, once again supporting H1. The results of the stability test of the mediation effect are shown in the model 3, after replacing the variable, COST is significantly negatively correlated with RD and CSR is significantly positively correlated with RD. Combined with the results of model 1 and model 2, it is further verified that debt financing cost has a partial intermediary role in the relationship between corporate social responsibility and enterprise innovation, and H2 is verified again. The test results of the moderating effect are shown from Model 4 to Model 6, where CSR*HHI in model 4 is significantly positive, which further verifies the H3a. The robustness test of moderated mediating effect shows that CSR*HHI in model 5 and COST*HHI in model 6 are both significantly negative, which again verifies H3b, indicating that the above related conclusions are robust.

**Table 6:** Robustness test—substitution variables.

Variables	Model 1	Model 2	Model 3	Model 4	Model 5	Model 6
	RD	COST	RD	RD	COST	RD
CSR	0.0119***	− 0.0115***	0.0102**	0.0253***	− 0.0136***	0.0221***
	(2.82)	(− 8.62)	(2.41)	(4.11)	(− 6.86)	(3.55)
HHI				0.1148***	− 0.0048	0.1395***
				(6.52)	(− 0.85)	(6.38)
CSRHHI				0.2345***	− 0.0348	0.2084***
				(3.23)	(− 1.48)	(2.84)
COST			− 0.1468***			− 0.1933***
			(− 4.90)			(− 4.30)
COSTHHI						− 1.1553*
						(− 1.95)
FS	0.0283***	− 0.0225***	0.0250***	0.0269***	− 0.0224***	0.0241***
	(8.52)	(− 21.23)	(7.39)	(8.22)	(− 21.13)	(7.20)
LEV	− 0.0446***	0.0191***	− 0.0418***	− 0.0438***	0.0190***	− 0.0413***
	(− 19.15)	(25.68)	(− 17.45)	(− 19.02)	(25.61)	(− 17.46)
AGE	− 0.0009***	0.0001***	− 0.0009***	− 0.0008***	0.0001***	− 0.0008***
	(− 14.66)	(3.40)	(− 14.51)	(− 14.19)	(3.26)	(− 14.03)
FSHR	− 0.0233***	− 0.0116***	− 0.0250***	− 0.0205***	− 0.0118***	− 0.0218***
	(− 9.60)	(− 14.97)	(− 10.21)	(− 8.51)	(− 15.22)	(− 8.99)
TQ	0.0077***	− 0.0002*	0.0077***	0.0072***	− 0.0002	0.0072***
	(22.17)	(− 1.89)	(22.10)	(20.86)	(− 1.53)	(20.82)
GROW	− 0.0035***	− 0.0016***	− 0.0037***	− 0.0040***	− 0.0016***	− 0.0043***
	(− 3.82)	(− 5.62)	(− 4.08)	(− 4.45)	(− 5.48)	(− 4.70)
ROA	− 0.0763***	− 0.0184***	− 0.0790***	− 0.0765***	− 0.0182***	− 0.0789***
	(− 10.79)	(− 8.16)	(− 11.15)	(− 10.89)	(− 8.03)	(− 11.21)
SIZE	0.0024***	0.0008***	0.0025***	0.0025***	0.0008***	0.0026***
	(6.34)	(6.66)	(6.65)	(6.78)	(6.57)	(7.06)
SOE	− 0.0015	− 0.0025***	− 0.0018**	− 0.0015*	− 0.0025***	− 0.0018**
	(− 1.61)	(− 8.74)	(− 2.02)	(− 1.66)	(− 8.75)	(− 2.05)
Constant	− 0.0196**	0.0104***	− 0.0181**	− 0.0079	0.0098***	− 0.0055
	(− 2.35)	(3.89)	(− 2.17)	(− 0.94)	(3.62)	(− 0.66)
Observations	10,953	10,953	10,953	10,953	10,953	10,953
R-squared	0.397	0.300	0.398	0.412	0.301	0.413
Year FE	YES	YES	YES	YES	YES	YES
Industry FE	YES	YES	YES	YES	YES	YES

***, ** and * respectively indicate that the parameter estimation is significant at the levels of 0.01, 0.05 and 0.1. The “t” value is in parentheses.

#### Bootstrap method

In this paper, we use the process plug-in designed by Hayes^38^. Besides, the bootstrap method under the 95% confidence interval is used, the sample size is set to 5000, and the mediation effect of debt financing cost and the moderating effect of market competition on the mediation effect is again examined. If the 95% confidence interval does not include 0, it is determined that the intermediary or direct effect exists significantly.

The test results for the mediation effect are shown in Table 7. It can be seen that none of the 95% confidence intervals for the above three effects include 0, which indicates that the total effect, direct effect and indirect effect are all significant. Consequently, there is a mediating effect. This result verifies H2, indicating that the above regression results for the mediating effect of debt financing cost are robust.

**Table 7:** Bootstrap results of the mediating effect.

Types of effect	Effect	BootSE	BootLLCI	BootULCI
Total effect	− 0.0039	0.002	0.001	0.008
Direct effect	− 0.0063	0.0021	− 0.0103	− 0.0021
Indirect effect	0.0024	0.0004	0.0017	0.0032

The test results of the moderated mediating effect are shown in Table 8. It can be seen that the first three columns in Table 8 show the indirect effect values of debt financing cost under different market competition levels. None of the three 95% confidence intervals for indirect effects contain 0, so indirect effect is considered significant. The fourth line of Table 8 examines the comparison of the mediating effect of debt financing cost under the low market competitiveness (Mean-1Sd) and the high market competitiveness (Mean + 1Sd), and it can be seen that the moderating effect of market competition is 0.002 and the confidence interval of 95% also excludes 0, indicating that the moderating effect is significant. With the increase of market competition, the intermediary effect of debt financing cost between corporate social responsibility and enterprise innovation increases. This result is consistent with the regression results above, and further verifies H3b, indicating that the above related conclusions are robust.

**Table 8:** Bootstrap results of moderated mediating effect.

	HHI	Effect	BootSE	BootLLCI	BootULCI
Indirect effect	Mean−1Sd	0.0013	0.0002	0.0009	0.0018
	Mean	0.0023	0.0004	0.0016	0.0031
	Mean + 1Sd	0.0034	0.0005	0.0024	0.0045
Indirect effect comparison		0.0239	0.0041	0.0163	0.0325

Mean refers to the average of the HHI index and 1Sd is a corresponding standard deviation.

#### The two-step systematic GMM method

Considering that enterprise innovation is a gradual process, and may have a dynamic lag effect on current innovation, we introduce the lag period I (L.RD) of the dependent variable (RD) as the independent variable, and further test the dynamic panel for basic regression in a robustness test.

In view of the fact that systematic GMM estimation can improve the efficiency of estimation while alleviating the limited sample bias and weak instrumentality of differential GMM, and the standard covariance matrix of the two-step estimation method can better deal with the problems of heteroscedasticity and autocorrelation, the two-step systematic GMM method is selected. Besides, systematic GMM estimation requires two tests to identify potential model setup biases, the autocorrelation test, the Sargan test, or the Hansen test. We choose the Arellano-Bond test and the Hansen Over-Recognition Constraint test.

The regression results are shown in Table 9. It can be seen that L.RD has a significant positive effect, and the core independent variable CSR still maintain good significance. Compared to the static panel results above, the symbols of the major variables did not change. The *P* value of AR(2) is greater than 0.1, indicating that there is no second-order autocorrelation. Moreover, the P value of Hansen is also greater than 0.1, indicating the null hypothesis that the tool variable is valid cannot be rejected. Consequently, it shows that the estimates in the previous study have strong robustness.

**Table 9:** The two-step systematic GMM method.

Variables	Model 1
	RD
L.RD	0.8842***
	(24.79)
CSR	0.0583***
	(2.66)
FS	0.0083
	(1.09)
LEV	0.0017
	(0.32)
AGE	0.0003*
	(1.95)
FSHR	− 0.0151***
	(− 2.59)
TQ	0.0000
	(0.00)
GROW	0.0026
	(1.01)
ROA	− 0.0220
	(− 0.79)
SIZE	− 0.0010
	(− 1.33)
SOE	− 0.0074***
	(− 2.77)
Constant	0.0245
	(0.01)
Observations	7,667
Year FE	YES
Industry FE	YES
AR(1)	0.000
AR(2)	0.546
Hansen(P)	0.135

***, ** and * respectively indicate that the parameter estimation is significant at the levels of 0.01, 0.05 and 0.1. The “t” value is in parentheses. AR(1), AR(2), and Hansen(P) are the *p*-values corresponding to the test statistic.

## Discussion

Taking the data of listed companies in China from 2016 to 2020 as a sample, this paper empirically examines the relationship between corporate social responsibility, debt financing cost and corporate innovation, and examines the moderating effect of market competition.

Different from the study of Chkir et al.^39^, CSR on corporate innovation is less pronounced in emerging countries. The results show that corporate social responsibility can promote R&D investment and improve the level of R&D innovation. CSR can reduce debt financing costs and ease financial pressure. Innovation requires high resource input and long cycle, and debt financing, as an important way for enterprises to seek funds for innovation and research and development, has a greater impact on enterprise innovation. Fulfilling social responsibilities reduces the cost of corporate debt financing, relieves financial pressure, and provides a financial basis for innovation investment.

In this paper, while studying the relationship between corporate social responsibility, debt financing cost and enterprise innovation, we add the fourth variable, namely the market competition degree, to explore the moderating effect of market competition. The construction method and testing process of the mediating effect model with regulation have been developed in the stream of research, however little research has been conducted on the moderating effect of market competition on the mediating effect model. The results suggest that the degree of market competition can strengthen the intermediary effect of debt financing cost in social responsibility and enterprise innovation, and the higher market competition degree, the stronger the intermediary effect of debt financing cost. In these many paths, the status of debt financing cost increases with the increase of market competition, that is, when the market competition is high, enterprises are more willing to invest low-cost debt financing from fulfilling social responsibilities into innovation, so as to maintain their market position and improve their core competitiveness.

## Conclusions and implications

### Conclusions

Based on stakeholder theory and information asymmetry theory, to generalize to a wide area, we take the data of listed companies in China as a sample, and analyze the relationship between corporate social responsibility and enterprise innovation from both theoretical and empirical aspects to examine moderated mediating effect. We find that corporate social responsibility can promote R&D investment and improve the level of R&D innovation. At the same time, debt financing cost has an mediating effect between corporate social responsibility and enterprise innovation. Corporate social responsibility reduces debt financing cost, thereby promoting the level of enterprise innovation, that is, the benefit of social responsibility to improve the level of enterprise innovation is partially achieved by reducing the cost of debt financing. The degree of market competition strengthens the positive effect of social responsibility on enterprise innovation, and the market competition degree can strengthen the mediating effect of debt financing cost. Therefore, we provide strong empirical evidence that can play an important role in improving corporate social responsibility as well as enterprise innovation.

### Management implications

#### From the corporate level

Enterprises should establish a positive and correct concept of social responsibility. In addition to assuming responsibilities to shareholders and creditors, enterprises should also devote themselves to environmental protection and social welfare undertakings, and achieve green safety of products and services. In terms of innovation funding, enterprises should recognize the role of debt financing costs in innovation, and actively find sufficient sources of funding for innovation. No matter what kind of market competition the enterprise is in, it should focus on long-term development.

#### From the government level

In formulating corresponding policies, the government may consider including the stakeholders of socially responsible enterprises into the scope of policy care. In terms of disclosure of social responsibility information, relevant departments should urge enterprises to disclose more standardized, specific and comparable social responsibility information.

Moreover, the government should increase support for innovative enterprises, relax financing channels, and promote the flow of social funds to enterprises. Besides, the government should strengthen the protection of property rights and help enterprises stay away from the infringement of innovation achievements. Finally, the government should strengthen the cultivation of innovative talents, actively introduce high-level talents from various places to meet the needs of enterprises for R&D personnel.

## Limitations and future research

This study has some limitations that open up avenues for future research. First, we select the data of China's A-share listed companies from 2016 to 2020 as a research sample, and does not involve companies listed in B-share and other cross-stock sectors, which may not be suitable for the research conclusions of this paper.

Second, in terms of the measurement method of CSR, this paper adopts the score of direct use of Hexun, which is relatively single. For enterprise innovation, this paper only examines the level of R&D investment. Consequently, more detailed measurement methods are needed for CSR and enterprise innovation in our future research.

## Supplementary Information

Supplementary Information.

## Data Availability

All the data used in this paper are openly available from Hexun network, Wind database, and China Stock Market and the Accounting Research (CSMAR) database and for more details on the website: https://www.hexun.com/https://www.wind.com.cn/https://www.gtarsc.com/.
