# Supplementary material for: Corporate social responsibility, debt financing cost and enterprise innovation
Source: Sci Rep. 2022 Dec 19;12:21909. doi: 10.1038/s41598-022-26076-3 (PMC9763416; doi:10.1038/s41598-022-26076-3)
Supplement: Supplementary file 1 — Supplementary Information. [file 41598_2022_26076_MOESM1_ESM.docx]

| **Variable type** | **Variable**  **symbol** | **Definition and description** |
| --- | --- | --- |
| Dependent variable | RD | The ratio of the R&D investment of the enterprise to the total assets at the end of the period. |
| Independent variable | CSR | Hexun Network scored Corporate Social Responsibility/100. |
| Mediating variable | COST | The ratio of interest expense for the current period to total liabilities at the end of the period. |
| Adjustment variable | HHI | The opposite of the concentration of the industry in which the enterprise is located. |
| Control variables | FS | The ratio of cash and cash equivalents to total assets. |
|  | LEV | The ratio of a company's total liabilities to total assets at the end of the period. |
|  | AGE | Observation year - the year in which the enterprise is listed. |
|  | FSHR | The ratio of number of shares held by the largest shareholder to total number of shares. |
|  | TQ | The ratio of enterprise Market Value to Total Assets. |
|  | GROW | (Current period operating income - previous period operating income) / previous period operating income. |
|  | ROA | The ratio of net profit to total assets at the end of the period. |
|  | SIZE | Natural logarithm of total assets at the end of the period. |
|  | SOE | State-owned companies are equal to 1, and private companies are equal to 0. |
|  | YEAR | Annual dummy variable. |
|  | IND | Industry dummy variable. |

**ST1.** Definition and description of control variables.

| **Variables** | **N** | **Mean** | **SD** | **Min** | **Median** | **Max** |
| --- | --- | --- | --- | --- | --- | --- |
| RD | 10953 | 0.0235 | 0.0196 | 0.0001 | 0.0201 | 0.1082 |
| CSR | 10953 | 0.2069 | 0.1037 | -0.0337 | 0.2104 | 0.6612 |
| COST | 10953 | 0.0168 | 0.0129 | 0 | 0.0153 | 0.0550 |
| HHI | 10953 | -0.0554 | 0.0437 | -0.2086 | -0.0409 | -0.0119 |
| FS | 10953 | 0.1643 | 0.1050 | 0.0148 | 0.1391 | 0.6179 |
| LEV | 10953 | 0.4173 | 0.1859 | 0.0741 | 0.4108 | 0.8645 |
| AGE | 10953 | 10.6153 | 7.3507 | 0.9699 | 8.7397 | 26.9973 |
| FSHR | 10953 | 0.4796 | 0.1486 | 0.1766 | 0.4733 | 0.8503 |
| TQ | 10953 | 1.9222 | 1.0976 | 0.8435 | 1.5851 | 7.2205 |
| GROW | 10953 | 0.1802 | 0.3733 | -0.4761 | 0.1151 | 2.3301 |
| ROA | 10953 | 0.0380 | 0.0624 | -0.2981 | 0.0381 | 0.1905 |
| SIZE | 10953 | 22.3415 | 1.2713 | 20.1193 | 22.1655 | 26.3653 |
| SOE | 10953 | 0.2660 | 0.4419 | 0 | 0 | 1 |

**ST2.** Descriptive statistics of variables.

| **Variables** | **RD** | **CSR** | **COST** | **HHI** | **FS** | **LEV** | **AGE** |
| --- | --- | --- | --- | --- | --- | --- | --- |
| RD | 1 |  |  |  |  |  |  |
| CSR | 0.023** | 1 |  |  |  |  |  |
| COST | -0.243*** | -0.203*** | 1 |  |  |  |  |
| HHI | 0.255*** | 0.00200 | -0.085*** | 1 |  |  |  |
| FS | 0.168*** | 0.154*** | -0.326*** | 0.069*** | 1 |  |  |
| LEV | -0.205*** | -0.125*** | 0.365*** | -0.131*** | -0.246*** | 1 |  |
| AGE | -0.235*** | -0.00600 | 0.189*** | -0.083*** | -0.071*** | 0.302*** | 1 |
| FSHR | -0.101*** | 0.176*** | -0.153*** | -0.107*** | 0.057*** | 0.020** | -0.148*** |
| TQ | 0.277*** | 0.056*** | -0.170*** | 0.134*** | 0.173*** | -0.303*** | -0.131*** |
| GROW | 0.022** | 0.139*** | -0.073*** | 0.020** | -0.00400 | 0.040*** | -0.085*** |
| ROA | 0.123*** | 0.574*** | -0.275*** | 0.038*** | 0.181*** | -0.298*** | -0.119*** |
| SIZE | -0.243*** | 0.187*** | 0.204*** | -0.155*** | -0.133*** | 0.519*** | 0.454*** |
| SOE | -0.187*** | 0.040*** | 0.057*** | -0.116*** | -0.024** | 0.257*** | 0.479*** |

**ST3.** Correlation Analysis of Variables. ***, ** and * respectively indicate that the parameter estimation is significant at the levels of 0.01, 0.05 and 0.1.

| **Variables** | **FSHR** | **TQ** | **GROW** | **ROA** | **SIZE** | **SOE** |
| --- | --- | --- | --- | --- | --- | --- |
| FSHR | 1 |  |  |  |  |  |
| TQ | -0.072*** | 1 |  |  |  |  |
| GROW | 0.039*** | 0.047*** | 1 |  |  |  |
| ROA | 0.177*** | 0.189*** | 0.228*** | 1 |  |  |
| SIZE | 0.163*** | -0.374*** | 0.035*** | 0.0130 | 1 |  |
| SOE | 0.136*** | -0.142*** | -0.048*** | -0.064*** | 0.360*** | 1 |

**ST3 (continued).** Correlation analysis of variables. ***, ** and * respectively indicate that the parameter estimation is significant at the levels of 0.01, 0.05 and 0.1.

| **Variables** | **Model 1** | **Model 2** | **Model 3** |
| --- | --- | --- | --- |
|  | **RD** | **COST** | **RD** |
| CSR | 0.0119***  (6.03) | -0.0115***  (-8.62) | 0.0098***  (5.00) |
| COST |  |  | -0.1787***  (-12.79) |
| FS | 0.0115***  (7.38) | -0.0225***  (-21.23) | 0.0075***  (4.74) |
| LEV | 0.0010  (0.87) | 0.0191***  (25.68) | 0.0044***  (3.91) |
| AGE | -0.0003***  (-11.39) | 0.0001***  (3.40) | -0.0003***  (-11.05) |
| FSHR | -0.0056***  (-4.88) | -0.0116***  (-14.97) | -0.0076***  (-6.68) |
| TQ | 0.0035***  (21.62) | -0.0002*  (-1.89) | 0.0035***  (21.55) |
| GROW | -0.0005  (-1.26) | -0.0016***  (-5.62) | -0.0008*  (-1.95) |
| ROA | 0.0160***  (4.81) | -0.0184***  (-8.16) | 0.0127***  (3.84) |
| SIZE | -0.0002  (-0.92) | 0.0008***  (6.66) | -0.0000  (-0.11) |
| SOE | -0.0000  (-0.07) | -0.0025***  (-8.74) | -0.0005  (-1.14) |
| Constant | 0.0012  (0.31) | 0.0104***  (3.89) | 0.0031  (0.79) |
| Observations | 10,953 | 10,953 | 10,953 |
| R-squared | 0.351 | 0.300 | 0.361 |
| Year FE | YES | YES | YES |
| Industry FE | YES | YES | YES |

**ST4.** Corporate social responsibility, debt financing cost and enterprise innovation. ***, ** and * respectively indicate that the parameter estimation is significant at the levels of 0.01, 0.05 and 0.1. The "t" value is in parentheses.

| **Variables** | **Model 4** | **Model 5** | **Model 6** |
| --- | --- | --- | --- |
|  | **RD** | **COST** | **RD** |
| CSR | 0.0182*** | -0.0136*** | 0.0145*** |
|  | (6.28) | (-6.86) | (4.99) |
| HHI | 0.0463*** | -0.0048 | 0.0692*** |
|  | (5.59) | (-0.85) | (6.77) |
| CSRHHI | 0.1092*** | -0.0348 | 0.0831** |
|  | (3.20) | (-1.48) | (2.42) |
| COST |  |  | -0.2322*** |
|  |  |  | (-11.08) |
| COSTHHI |  |  | -1.0816*** |
|  |  |  | (-3.91) |
| FS | 0.0109*** | -0.0224*** | 0.0071*** |
|  | (7.08) | (-21.13) | (4.52) |
| LEV | 0.0013 | 0.0190*** | 0.0046*** |
|  | (1.22) | (25.61) | (4.15) |
| AGE | -0.0003*** | 0.0001*** | -0.0003*** |
|  | (-10.92) | (3.26) | (-10.55) |
| FSHR | -0.0043*** | -0.0118*** | -0.0062*** |
|  | (-3.85) | (-15.22) | (-5.50) |
| TQ | 0.0033*** | -0.0002 | 0.0033*** |
|  | (20.40) | (-1.53) | (20.38) |
| GROW | -0.0008* | -0.0016*** | -0.0011** |
|  | (-1.80) | (-5.48) | (-2.51) |
| ROA | 0.0158*** | -0.0182*** | 0.0126*** |
|  | (4.78) | (-8.03) | (3.82) |
| SIZE | -0.0001 | 0.0008*** | 0.0000 |
|  | (-0.60) | (6.57) | (0.21) |
| SOE | -0.0000 | -0.0025*** | -0.0005 |
|  | (-0.09) | (-8.75) | (-1.19) |
| Constant | 0.0060 | 0.0098*** | 0.0087** |
|  | (1.52) | (3.62) | (2.23) |
| Observations | 10,953 | 10,953 | 10,953 |
| R-squared | 0.365 | 0.301 | 0.374 |
| Year FE | YES | YES | YES |
| Industry FE | YES | YES | YES |

**ST5.** The moderation effect of market competition degree. ***, ** and * respectively indicate that the parameter estimation is significant at the levels of 0.01, 0.05 and 0.1. The "t" value is in parentheses.

| **Variables** | **Model 1** | **Model 2** | **Model 3** | **Model 4** | **Model 5** | **Model 6** |
| --- | --- | --- | --- | --- | --- | --- |
|  | **RD** | **COST** | **RD** | **RD** | **COST** | **RD** |
| CSR | 0.0119*** | -0.0115*** | 0.0102** | 0.0253*** | -0.0136*** | 0.0221*** |
|  | (2.82) | (-8.62) | (2.41) | (4.11) | (-6.86) | (3.55) |
| HHI |  |  |  | 0.1148*** | -0.0048 | 0.1395*** |
|  |  |  |  | (6.52) | (-0.85) | (6.38) |
| CSRHHI |  |  |  | 0.2345*** | -0.0348 | 0.2084*** |
|  |  |  |  | (3.23) | (-1.48) | (2.84) |
| COST |  |  | -0.1468*** |  |  | -0.1933*** |
|  |  |  | (-4.90) |  |  | (-4.30) |
| COSTHHI |  |  |  |  |  | -1.1553* |
|  |  |  |  |  |  | (-1.95) |
| FS | 0.0283*** | -0.0225*** | 0.0250*** | 0.0269*** | -0.0224*** | 0.0241*** |
|  | (8.52) | (-21.23) | (7.39) | (8.22) | (-21.13) | (7.20) |
| LEV | -0.0446*** | 0.0191*** | -0.0418*** | -0.0438*** | 0.0190*** | -0.0413*** |
|  | (-19.15) | (25.68) | (-17.45) | (-19.02) | (25.61) | (-17.46) |
| AGE | -0.0009*** | 0.0001*** | -0.0009*** | -0.0008*** | 0.0001*** | -0.0008*** |
|  | (-14.66) | (3.40) | (-14.51) | (-14.19) | (3.26) | (-14.03) |
| FSHR | -0.0233*** | -0.0116*** | -0.0250*** | -0.0205*** | -0.0118*** | -0.0218*** |
|  | (-9.60) | (-14.97) | (-10.21) | (-8.51) | (-15.22) | (-8.99) |
| TQ | 0.0077*** | -0.0002* | 0.0077*** | 0.0072*** | -0.0002 | 0.0072*** |
|  | (22.17) | (-1.89) | (22.10) | (20.86) | (-1.53) | (20.82) |
| GROW | -0.0035*** | -0.0016*** | -0.0037*** | -0.0040*** | -0.0016*** | -0.0043*** |
|  | (-3.82) | (-5.62) | (-4.08) | (-4.45) | (-5.48) | (-4.70) |
| ROA | -0.0763*** | -0.0184*** | -0.0790*** | -0.0765*** | -0.0182*** | -0.0789*** |
|  | (-10.79) | (-8.16) | (-11.15) | (-10.89) | (-8.03) | (-11.21) |
| SIZE | 0.0024*** | 0.0008*** | 0.0025*** | 0.0025*** | 0.0008*** | 0.0026*** |
|  | (6.34) | (6.66) | (6.65) | (6.78) | (6.57) | (7.06) |
| SOE | -0.0015 | -0.0025*** | -0.0018** | -0.0015* | -0.0025*** | -0.0018** |
|  | (-1.61) | (-8.74) | (-2.02) | (-1.66) | (-8.75) | (-2.05) |
| Constant | -0.0196** | 0.0104*** | -0.0181** | -0.0079 | 0.0098*** | -0.0055 |
|  | (-2.35) | (3.89) | (-2.17) | (-0.94) | (3.62) | (-0.66) |
| Observations | 10,953 | 10,953 | 10,953 | 10,953 | 10,953 | 10,953 |
| R-squared | 0.397 | 0.300 | 0.398 | 0.412 | 0.301 | 0.413 |
| Year FE | YES | YES | YES | YES | YES | YES |
| Industry FE | YES | YES | YES | YES | YES | YES |

**ST6.** Robustness test——substitution variables. ***, ** and * respectively indicate that the parameter estimation is significant at the levels of 0.01, 0.05 and 0.1. The "t" value is in parentheses.

| **Types of effect** | **Effect** | **BootSE** | **BootLLCI** | **BootULCI** |
| --- | --- | --- | --- | --- |
| Total effect | -0.0039 | 0.002 | 0.001 | 0.008 |
| Direct effect | -0.0063 | 0.0021 | -0.0103 | -0.0021 |
| Indirect effect | 0.0024 | 0.0004 | 0.0017 | 0.0032 |

**ST7.** Bootstrap results of the mediating effect.

|  | **HHI** | **Effect** | **BootSE** | **BootLLCI** | **BootULCI** |
| --- | --- | --- | --- | --- | --- |
| Indirect effect | Mean-1Sd | 0.0013 | 0.0002 | 0.0009 | 0.0018 |
|  | Mean | 0.0023 | 0.0004 | 0.0016 | 0.0031 |
|  | Mean+1Sd | 0.0034 | 0.0005 | 0.0024 | 0.0045 |
| Indirect effect comparison |  | 0.0239 | 0.0041 | 0.0163 | 0.0325 |

**ST8.** Bootstrap results of moderated mediating effect. Mean refers to the average of the HHI index and 1Sd is a corresponding standard deviation.

| **Variables** | **Model 1** |
| --- | --- |
|  | **RD** |
| L.RD | 0.8842*** |
|  | (24.79) |
| CSR | 0.0583*** |
|  | (2.66) |
| FS | 0.0083 |
|  | (1.09) |
| LEV | 0.0017 |
|  | (0.32) |
| AGE | 0.0003* |
|  | (1.95) |
| FSHR | -0.0151*** |
|  | (-2.59) |
| TQ | 0.0000 |
|  | (0.00) |
| GROW | 0.0026 |
|  | (1.01) |
| ROA | -0.0220 |
|  | (-0.79) |
| SIZE | -0.0010 |
|  | (-1.33) |
| SOE | -0.0074*** |
|  | (-2.77) |
| Constant | 0.0245 |
|  | (0.01) |
| Observations | 7,667 |
| Year FE | YES |
| Industry FE | YES |
| AR(1) | 0.000 |
| AR(2) | 0.546 |
| Hansen(P) | 0.135 |

**ST9.** The two-step systematic GMM method. ***, ** and * respectively indicate that the parameter estimation is significant at the levels of 0.01, 0.05 and 0.1. The "t" value is in parentheses. AR(1), AR(2), and Hansen(P) are the p-values corresponding to the test statistic.
